# Silicon Priming Created an Enhanced Tolerance in Alfalfa (*Medicago sativa* L.) Seedlings in Response to High Alkaline Stress

**DOI:** 10.3389/fpls.2018.00716

**Published:** 2018-05-29

**Authors:** Duo Liu, Miao Liu, Xiao-Long Liu, Xian-Guo Cheng, Zheng-Wei Liang

**Affiliations:** ^1^Northeast Institute of Geography and Agroecology, Chinese Academy of Sciences, Changchun, China; ^2^College of Resources and Environment, University of Chinese Academy of Sciences, Beijing, China; ^3^Lab of Plant Nutrition Molecular Biology, Institute of Agricultural Resources and Regional Planning, Chinese Academy of Agricultural Sciences, Beijing, China

**Keywords:** alfalfa, alkaline stress, silicon priming, tolerance, osmolytes

## Abstract

Alkaline stress as a result of higher pH usually triggers more severe physiological damage to plants than that of saline stress with a neutral pH. In the present study, we demonstrated that silicon (Si) priming of alfalfa (*Medicago sativa* L.) seedlings increased their tolerance to high alkaline stress situations. Gongnong No. 1 seedlings were subjected to alkaline stress simulated by 25 mM Na_2_CO_3_ (pH 11.2). Alkaline stress greatly decreased the biomass and caused severe lodging or wilting of alfalfa seedlings. In contrast, the application of Si to alfalfa seedlings 36 h prior to the alkaline treatment significantly alleviated the damage symptoms and greatly increased the biomass and chlorophyll content. Because of being concomitant with increasing photosynthesis and water use efficiency, decreasing membrane injury and malondialdehyde content, and increasing peroxidase and catalase ascorbate activities in alfalfa leaves, thereby alleviating the triggered oxidative damage by alkaline stress to the plant. Furthermore, Si priming significantly decreased the accumulation of protein and proline content in alfalfa, thus reducing photosynthetic feedback repression. Si priming significantly accumulated more Na in the roots, but led to a decrease of Na accumulation and an increase of K accumulation in the leaves under alkaline stress. Meanwhile, Si priming decreased the accumulation of metal ions such as Mg, Fe, Mn, and Zn in the roots of alfalfa seedlings under alkaline stress. Collectively, these results suggested that Si is involved in the metabolic or physiological changes and has a potent priming effect on the alkaline tolerance of alfalfa seedlings. The present study indicated that Si priming is a new approach to improve the alkaline tolerance in alfalfa and provides increasing information for further exploration of the alkaline stress response at the molecular level in alfalfa.

## Introduction

Salinity-alkalinity stress is an adverse obstacle in the production of agricultural crops and severely affects the growth of plants. Globally, it has been estimated that approximately 3.97 × 10^8^ ha of land is affected by salt and 4.34 × 10^8^ ha is alkaline (Martinez-Beltran and Manzur, 2005). However, alkaline soils (high pH from 8.5 to 11) endanger crop production more than soils containing excess salt (Guo et al., 2017; Wei et al., 2017). To date, the effects of salt stress on plants have been widely reported (Sun et al., 2016; Shu et al., 2017; Zhu et al., 2017). In contrast, fewer studies have examined alkaline stress (Lv et al., 2015; Guo et al., 2017; Zhang et al., 2017). Understanding how plants respond to alkaline stress is essential for improving the alkaline tolerance of plants.

Alfalfa (*Medicago sativa* L.) has been widely planted because of its high biomass production and high nutritional quality and is identified as a leading forage crop that is known as the “Queen of the forages” by Kumar (2011). To meet the increasing requirements for feeding livestock, the planting areas of alfalfa have been expanded in northeast China. However, alkaline stress is a major limiting factor in alfalfa production in these areas (Peng et al., 2008). Hence, it is of great importance to develop practical and efficient approaches to enhance the resistance of alfalfa seedlings to alkaline stress, and thereby improve alfalfa productivity.

Plants can successfully tolerate various stresses after pretreatment with a series of chemical compounds, which is referred to as “priming,” and the chemically treated plants are considered to be in a “primed” state (Beckers and Conrath, 2007; Aranega-Bou et al., 2014; Savvides et al., 2016). Previous studies have shown that silicon (Si) priming plays a positive regulatory role in enhancing the adaptability of diverse plant species to various stresses (Wang and Han, 2007; Guntzer et al., 2012; Li et al., 2015; Kabir et al., 2016). However, these studies were performed by adding Si simultaneously with the stress, and the pH of the culture solution usually ranged from 6.0 to 6.5. These studies did not refer to priming only and the notable feature of high pH during alkaline stress was also neglected. At present, only one study has shown that Si priming enhanced alkaline tolerance in maize by soaking seeds with Si solution (Abdel Latef and Tran, 2016); however, no physiological responses in alfalfa from Si priming in response to alkaline stress were reported and the mechanisms of Si priming still remains unknown.

The present study aimed to gain insights into the physiological metabolites and oxidative defense system of Si priming to alkaline stress to better understand the physiological modulation of Si priming involved in the adaptation of alfalfa to alkaline stress.

## Materials and Methods

### Plant Material and Growth Conditions

The local alfalfa cultivar (Gongnong No. 1) was used in the present study. Alfalfa seeds were sterilized with a solution containing 6% sodium hypochlorite for 10 min and germinated by culture in vermiculite for 10 days. The growing seedlings were secured onto the holes of a foam plate and cultured in black plastic containers containing 1000 ml of 1/2 Hoagland’s solution. Black containers were used to specifically minimize irradiation induced by heating and to avoid algal growth. The containers were placed in a growth chamber at temperatures of 25°C day/20°C night, and a 12 h photoperiod with 70% humidity and 350 μmol m^-2^ s^-1^ light intensity (Zhang et al., 2017).

### Determination of Optimum Dosage of Si Addition

A preliminary culture experiment was performed to determine the optimum addition concentration of Na_2_CO_3_ based on the plant growth inhibition (Supplementary Figure S1). To obtain the optimum dosage of Si addition, 30-day-old alfalfa seedlings were cultured in solutions containing Si based on a previous method (Liang et al., 2006). Briefly, alfalfa seedlings were cultured in pure distilled water under nutrient starvation for 24 h, and then transferred into Hoagland’s solutions containing different Si concentrations ranging from 0.075 to 3.75 mM, and continuously cultured for 24 h. Finally, the Si amounts in the residual solutions were measured by the molybdenum blue colorimetric method to determine the adsorption rate peaks of Si in alfalfa.

### Stress Treatment

We used Na_2_CO_3_ at 25 mM (pH = 11.2, EC_1:5_ = 3.7 mS cm^-1^) to simulate alkaline stress in a hydroponics experiment. The EC_1:5_ and pH of the solutions were measured using a DDS-12 conductivity meter (Lida In., Shanghai, China) and PHS-25 pH meter (Baiyuan In., Beijing, China), respectively. For Si priming pretreatment, the same number of 30-day-old alfalfa seedlings were separately transplanted in the black plastic pots containing 0 (control) or 2.25 mM Na_2_SiO_3_⋅9H_2_O to perform Si priming of the root, and further cultured for 36 h, and followed a further culture in black plastic containers containing 1000 ml of 1/2 Hoagland’s solution in the presence or in the absence of 25 mM Na_2_CO_3_ for 48 h under the same culture conditions as described in “Plant Material and Growth Conditions” above.

### Measurement of Morphological Parameters of Roots and Biomass of Alfalfa Seedlings

A total of 5 plants of alfalfa seedlings for each replication was randomly selected from the 25 seedlings with 5 replications in each treatment after 48 h of alkaline stress. The roots of each plant seedling were scanned using an Epson Expression 10000XL (Epson America Inc., Long Beach, CA, United States). The resulting images were digitized using the WinRHIZO program according to the instructions of the manufacturer (Regent Instruments Canada Inc., Ville de Québec, QC, Canada), and the root parameters, total root length, total root surface area, total root volume, average root diameter, and root number of forks were determined. After scanning, the seedlings were cut and divided into aboveground parts and roots. The fresh weight (FW) of alfalfa seedlings was estimated after washing with deionized water and blotting on paper towel. These samples were then dried in a forced-air-driven oven at 105°C for 2 h, followed by 70°C until maintaining a stable mass prior to the determination of dry mass (Zhang et al., 2017).

### Measurement of Photosynthetic Attributes

Photosynthetic rate (Pn), stomatal conductance (Gs), and transpiration rate (Tr) in the first fully expanded leaves were recorded using a Li-6400 Portable Photosynthesis System (Li-Cor Inc., Lincoln, NE, United States) between 10:00 AM and 12:00 AM. Water use efficiency (WUE) was calculated by the changes in the photosynthetic and transpiration rates.

### Measurement of Chlorophyll Content

A total of 0.5 g of fresh fully expanded leaves was extracted with 80% acetone, and the contents of chlorophyll a and chlorophyll b were determined at 663 and 645 nm, respectively, by an UV-visible spectrophotometer (UV-2700, Shimadzu, Kyoto, Japan). Total chlorophyll content was calculated using the following formula: 8.02 OD_663__nm_ + 20.21 OD_645__nm_V/1000W.

### Measurement of Protein, Proline, and Soluble Sugar Content

The protein content in fresh leaves was measured colorimetrically at 595 nm based on the formation of protein-dye complex (the binding of Coomassie Brilliant Blue G-250 to protein) using bovine serum albumin as the standard (Bradford, 1976).

The proline content in fresh leaves was determined following the methodology described by Bates et al. (1973). In brief, approximately 0.5 g of fresh leaves were extracted by a boiling solution of 3% (w/v) 5-sulfosalicylic acid for 10 min and cooled to room temperature (25–28°C). Proline content in the extracts was determined based on the absorbance at 520 nm. For a calculation of proline content, a standard curve representing different appropriate proline contents was established.

Soluble sugar content in leaves was measured according to the method of Irigoyen et al. (1992) with slight modification. Standard solutions were prepared by the solutions, respectively, containing 0, 20, 40, 60, 80, and 100 g ml^-1^of sucrose. A total of 0.5 ml standard solution of each solution was supplemented by adding 0.5 ml of anthrone ethyl acetate solution and 5 ml of sulfuric acid, and then boiled in water bath for 1 min. After cooling to room temperature (25–28°C), the absorbance at 630 nm was determined by a UV-visible spectrophotometer and the standard curve was obtained. A total of 0.3 g of fresh leaf segments were put into a centrifuge tube containing 10 ml of distilled water, and boiled for 30 min and the resulting extracts were filtered through two layers of cheesecloth. An aliquot of 0.5 ml filtrate was taken and subjected to determining absorbance changes at 630 nm as described above. The soluble sugar contents were estimated according to the standard curve. The FW of leaves was converted to dry weight (DW) by subtracting water content, and the sugar contents were expressed as mg equivalents of sucrose per g DW.

### Measurement of Membrane Injury (MI) and Malondialdehyde (MDA) Content

Fresh leaves were collected for measurement of the MI by electrolyte leakage using a previously described method (Li et al., 2012). Briefly, a total of 2 g of fresh leaves were submerged in 10 ml of deionized water in a conical tube of 15 ml and maintained at 20°C for 1 h, and the EC of effusion was measured (EC_1_). The same weight of fresh leaf tissues was boiled in water bath for 40 min and cooled to 20°C, and the EC of the effusion was measured (EC_2_). MI was evaluated by the formula MI (%) = EC_1_/EC_2_ × 100%.

The MDA content in the leaves was determined by the thiobarbituric acid reaction based on the method described by Heath and Packer (1968). A total of 0.1 g of fresh leaves were homogenized in 1 ml of 50 mM phosphate buffer solution (pH 7.8) using a bench-top ball-mill (Scientz-48, Ningbo Scientz Biotechnology Co., Ltd., Ningbo, China) at 50 Hz for 30 s, and the extract was centrifuged at 4830 × g at 4°C for 15 min. Subsequently, 400 ml of the supernatant was mixed with 1 ml of 0.5% thiobarbituric acid and the mixture was heated at 100°C for 20 min, then cooled and centrifuged at 7888 × g again and the absorbances of the harvested supernatant was measured at 532, 600, and 450 nm to determine the MDA content by the formula MDA = 6.452 × (A_532_ – A_600_) – 0.56 × A_450_.

### Measurement of Antioxidant Enzyme Activities

A total of 0.1 g of fresh leaves was placed into a 2 ml tube and frozen in liquid nitrogen, and then homogenized in 1 ml of 50 mM phosphate buffer solution (pH 7.8) using a bench-top ball-mill at 50 Hz for 30 s. The homogenate was centrifuged at 4830 × g at 4°C for 15 min, and the supernatant was used for enzymatic assays of superoxide dismutase (SOD), catalase ascorbate (CAT), and peroxidase (POD) activities.

SOD (EC 1.15.1.1) activity was assayed using the nitro blue tetrazolium method (Giannopolitis and Ries, 1977). One unit of SOD was defined as the required amount for the enzyme reaction depending on 50% inhibition of nitro blue tetrazolium reduction based on the measurements at 560 nm.

Catalase ascorbate (EC 1.11.1.6) activity was monitored based on the method described by Aebi (1984) and assayed by the decline in absorbance per minute at 240 nm as a consequence of H_2_O_2_ consumption.

Peroxidase (EC1.11.1.7) activity was determined by assessing the rate of guaiacol oxidation in the presence of H_2_O_2_. One unit of POD was defined as the increase in absorbance per minute at 470 nm (Klapheck et al., 1990).

To assay ascorbate peroxidase (APX) activity, the samples were first extracted with 1 ml of 50 mM phosphate buffer solution (pH 7.0) containing 1 mM AsA and 1 mM ethylenediaminetetraacetic acid, then homogenized at 50 Hz for 30 s, and the homogenate centrifuged at 4830 × g at 4°C for 15 min. Subsequently, the supernatant was mixed with phosphate buffer solution (pH 7.0) containing 15 mM AsA and 0.3 mM H_2_O_2_. The reaction mixture was analyzed at 290 nm. One unit of APX was defined as the variable quantity of absorbance at 290 nm per minute.

### Measurement of Ion Content

A total of 0.1 g of dried powder sample was digested completely with a mixture of HNO_3_ and HClO_4_ (v/v = 2:1) and diluted to 50 ml. The total contents of K, Na, Ca, Fe, Mg, Cu, Zn, P, and S in the leaves, stems, and roots were determined by flame emission spectrometry (FP6410; Shanghai Precision Instrument Co., Ltd., China).

### Statistical Analyses

All data were represented by five replications and statistical analyses were performed using the statistical software program SPSS 19.0 (IBM Co., Armonk, NY, United States). Based on the analysis of variance (ANOVA) results, a Duncan’s multiple range test was performed (*P* < 0.05). Sigma Plot version 10.0 was used for graphical data presentation (Systat Software Inc., San Jose, CA, United States).

## Results

### Silicon Priming Alleviated Alkaline Damage During Alfalfa Growth

**FIGURE 1:**
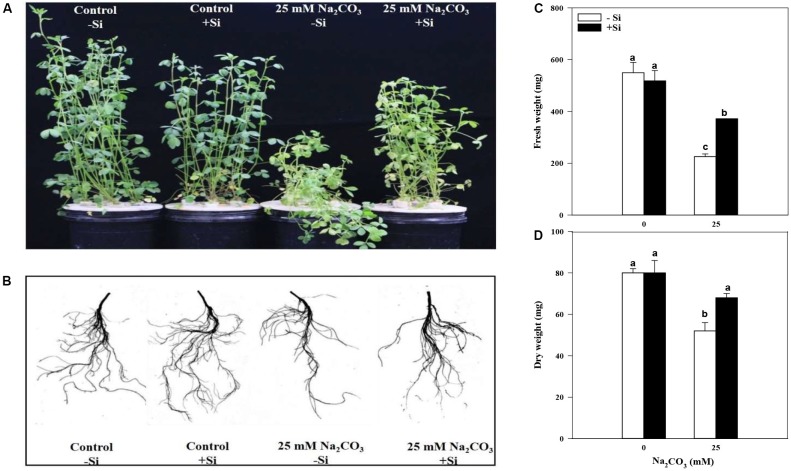
Image of Phenotype **(A)**, root morphological structures **(B)**, aboveground fresh weight **(C)**, and aboveground dry weight **(D)** of alfalfa seedlings under alkaline stress. -Si = pretreatment without Si; +Si = pretreatment with Si. Values are means ± SE, *n* = 25. Different letters denote significant difference (*P* < 0.05) based on Duncan’s test.

**FIGURE 2:**
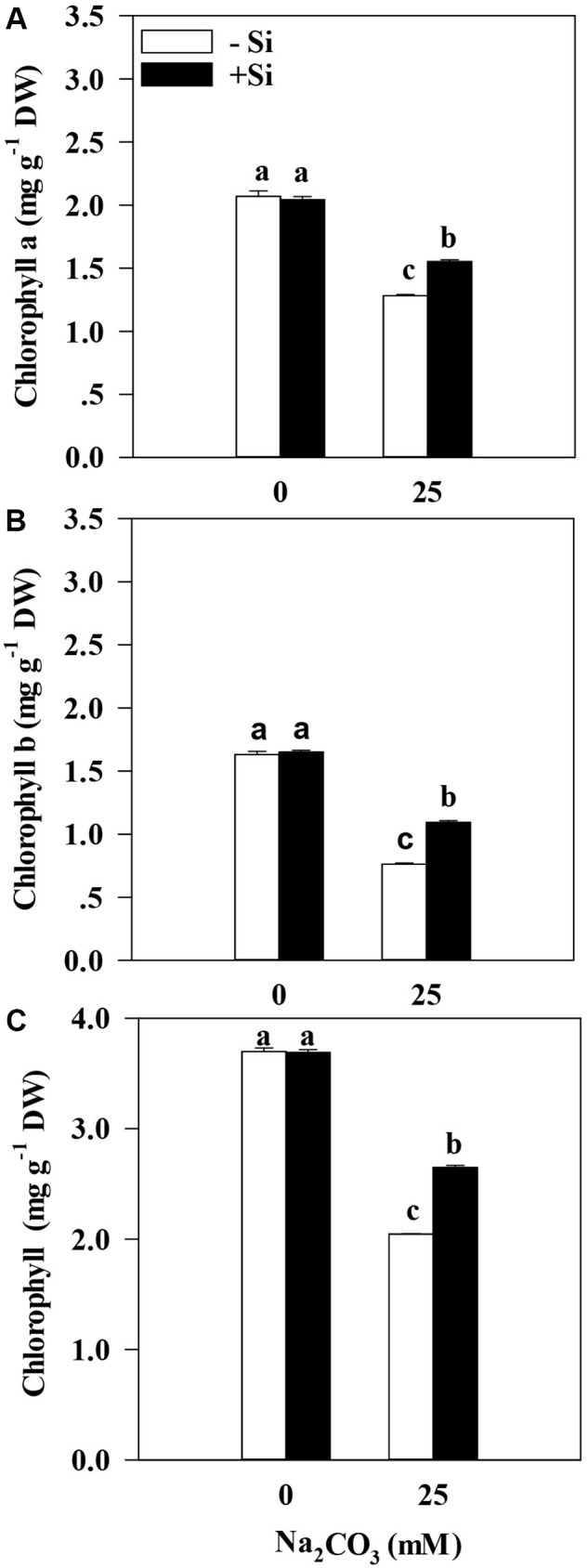
Chlorophyll a content **(A)**, Chlorophyll b content **(B)**, and total chlorophyll content **(C)** in the leaves of alfalfa seedlings under Si priming in response to alkaline stress. –Si = pretreatment without Si; +Si = pretreatment with Si. Values are means ± SE, *n* = 25. Different letters denote significant difference (*P* < 0.05) based on Duncan’s test.

**FIGURE 3:**
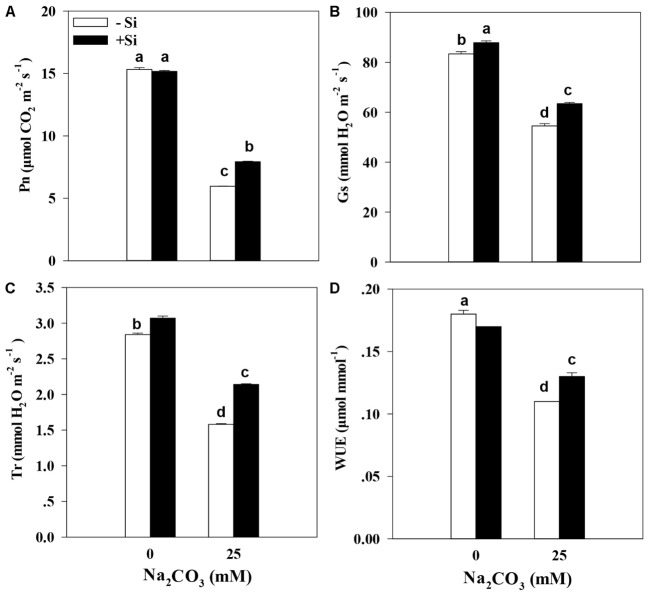
Net photosynthetic rate (Pn) **(A)**, stomatal conductance (Gs) **(B)**, transpiration (Tr) **(C)**, and water use efficiency (WUE) **(D)** in the leaves of alfalfa seedlings under Si priming in response to alkaline stress. –Si = pretreatment without Si; +Si = pretreatment with Si. Values are means ± SE, *n* = 25. Different letters denote significant difference (*P* < 0.05) based on Duncan’s test.

**FIGURE 4:**
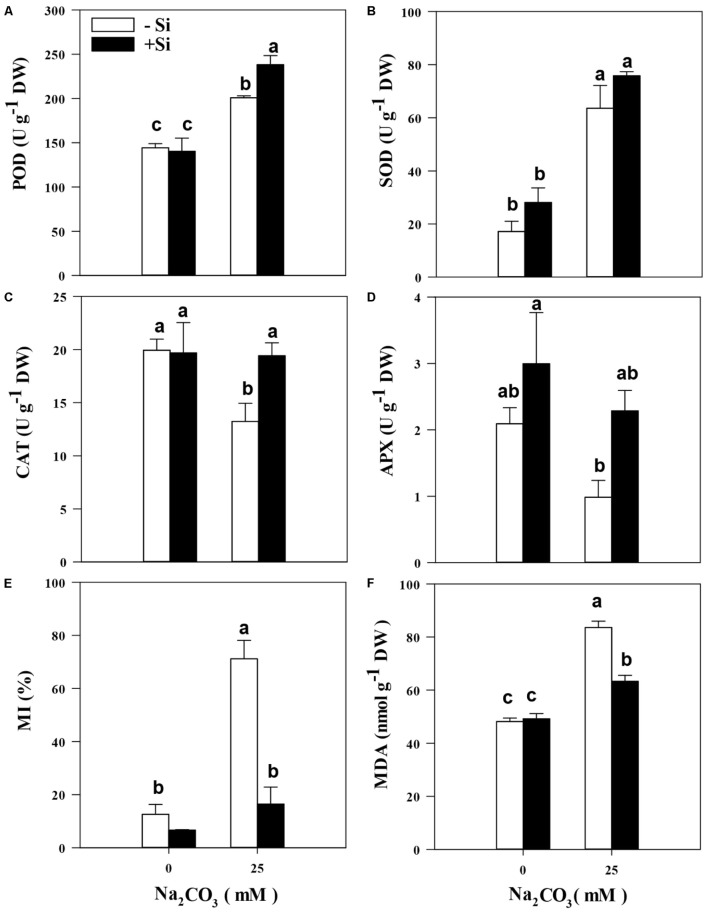
Peroxidase (POD) activity **(A)**, superoxide (SOD) activity **(B)**, catalase (CAT) activity **(C)**, ascorbate peroxidase (APX) activity **(D)**, membrane injury (MI) **(E)**, and malondialdehyde content (MDA) **(F)** in the leaves of alfalfa seedlings under Si priming in response to alkaline stress. –Si = pretreatment without Si; +Si = pretreatment with Si. Values are means ± SE, *n* = 25. Different letters denote significant difference (*P* < 0.05) based on Duncan’s test.

**FIGURE 5:**
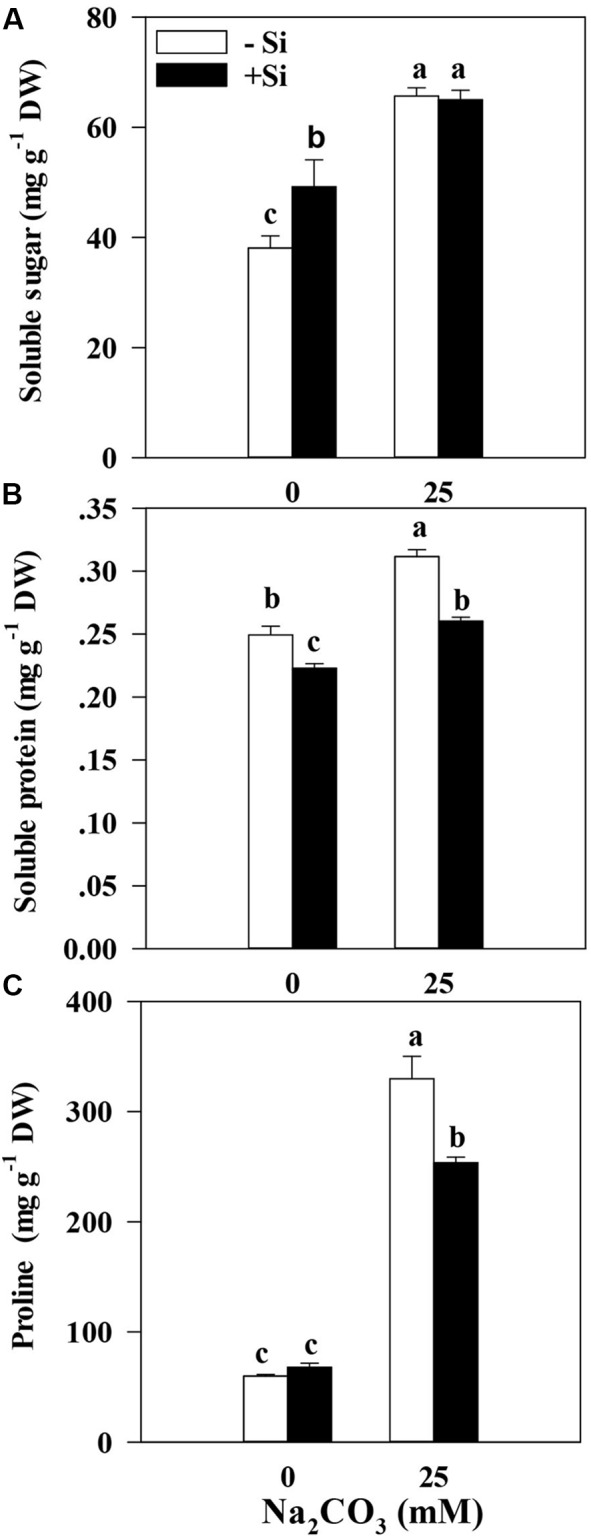
Soluble sugar content (sucrose) **(A)**, soluble protein content **(B)**, and proline content **(C)** in the leaves of alfalfa seedlings under Si priming in response to alkaline stress. –Si = pretreatment without Si; +Si = pretreatment with Si. Values are means ± SE, *n* = 25. Different letters denote significant difference (*P* < 0.05) based on Duncan’s test.

To determine the phenotypic changes from Si priming in response to alkaline stress, plant biomass was measured. As shown in **Figure 1A**, Si priming alleviated alkaline damage to the alfalfa plants. In particular, when alfalfa seedlings with Si pretreatment were cultured in the 25 mM Na_2_CO_3_ solution, did not exhibit apparent lodging compared to the treatment without Si pretreatment, and the aboveground biomass of alfalfa seedlings were significantly increased (**Figures 1C,D**). This indicates that Si priming enhanced alkaline tolerance in alfalfa. Generally, alkaline stress first incarnates morphological changes in the roots and influences its architecture. Thus, we analyzed the root developmental parameters by scanning the roots of alfalfa seedlings (**Figure 1B**). Si priming led to significant increase in root DW (74.2%) and average root diameter (20.2%) under alkaline stress compared to the alkaline stress treatment without Si priming (**Table 1**). In addition, FW, total root length, total root surface area, total root volume and root fork number also showed an increasing tendency despite of having no statistically significant differences. However, no significant effects of Si priming on plant growth and physiological parameters were observed under unstressed conditions (**Figures 1–5** and **Tables 1–3**).

**Table 1:** Effects of Si priming on morphological parameters of roots in alfalfa seedlings under alkaline stress.

Na_2_CO_3_ (mM)		Fresh weight (mg)	Dry weight (mg)	Root length (cm)	Surface area (cm^2^)	Average diameter (mm)	Volume (cm^3^)	Number of forks
0	-Si	353.54 ± 34.28^a^	47.58 ± 3.94^a^	1343 ± 159^a^	223.61 ± 22.81^a^	0.53 ± 0.01^c^	2.97 ± 0.27^b^	3848 ± 382^a^
	+Si	346.31 ± 29.72^a^	31.75 ± 2.69^bc^	1275 ± 234^a^	212.60 ± 37.66^a^	0.53 ± 0.01^b^	2.83 ± 0.49^ab^	4846 ± 551^a^
25	-Si	331.49 ± 10.11^a^	23.83 ± 1.30^c^	1201 ± 13^a^	182.36 ± 6.62^a^	0.48 ± 0.02^b^	2.22 ± 0.15^ab^	4510 ± 797^a^
	+Si	423.20 ± 42.55^a^	41.51 ± 8.54^ab^	1266 ± 115^a^	230.75 ± 19.74^a^	0.58 ± 0.01^a^	3.35 ± 0.27^a^	5595 ± 774^a^


***-**Si, pretreatment without Si; +Si, pretreatment with Si. Values are means ± SE, *n* = 25. Different letters denote significant difference (*P* < 0.05) based on Duncan’s test.*

### Silicon Priming Improved the Photosynthetic Process

Inhibition of alkaline stress-triggered in alfalfa growth usually affects the photosynthetic process in plants. Therefore, we performed analyses of photosynthetic parameters, which are closely associated with photosynthesis, in response to alkaline stress. Results showed that the pretreatment culture with Si did not alter accumulation profiles of chlorophyll a, chlorophyll b, and total chlorophyll in alfalfa under the favorable culture conditions (**Figure 2**). Under alkaline stress, the chlorophyll a content was 38.0% lower, the chlorophyll b content was 53.2% lower, and the total chlorophyll content was 44.7% lower than that of the control without Si priming (**Figures 2A–C**). Additionally, the treatment with Si priming significantly increased chlorophyll a (21.1%), chlorophyll b (43.3%), and total chlorophyll (29.5%) contents in alfalfa seedlings than the treatment without Si pretreatment under alkaline stress (*P* < 0.05) (**Figures 2A–C**). Chlorophyll accumulation profiles directly influence the photosynthesis and WUE (**Figure 3**). Measurements showed that alkaline stress resulted in a decrease of 61.3% in Pn, 34.6% in Gs, 44.4% in Tr, and 39.7% in WUE in alfalfa seedlings that were not treated with Si comparing to the control (**Figures 3A–D**). However, the Si priming treatment led to an increase of Pn (33.8%), Gs (16.4%), Tr (35.6%), and WUE (15.9%) in alfalfa seedlings than those in the treatment without Si priming under alkaline stress (*P* < 0.05) (**Figures 3A–D**).

### Silicon Priming Enhanced Activities of Antioxidant Enzymes and Alleviated Cell Membrane Damage

Alkaline stress often alters the activities of antioxidant enzymes, and thus affects the ability of plant oxidation resistance. Measurements showed that the POD and SOD activities in alfalfa seedlings of the Si priming treatment were 68.1 and 240.1% higher than those of the control under alkaline conditions, respectively (**Figures 4A,B**). However, the CAT and APX activities in alfalfa seedlings of the Si priming treatment were 39.9 and 73.4% lower, than those of the control under alkaline conditions, respectively (**Figures 4C,D**). Notably, the treatment with Si priming produced higher POD (18.6%), SOD (21.8%), CAT (46.7%), and APX (177.5%) activities than the treatment without Si priming under alkaline stress (**Figures 4A–D**). As shown in **Figure 4**, alkaline stress caused higher cell membrane damage increase of 463.9% in MI (**Figure 4E**) and increase of 57.0% in MDA (**Figure 4F**) comparing to the control treatment. However, Si priming produced significantly lower MI (76.9%) and MDA (19.6%) compared with the treatment without Si priming under alkaline stress (*P* < 0.05).

### Silicon Priming Altered Accumulation Profiles of Osmolytes and Ions in Alfalfa Under Alkaline Stress

Accumulation profiles of compatible solutes are closely associated with the levels of osmotic potentials, which affect the osmotic balance in plants. Therefore, we evaluated the accumulation profiles of compatible solutes such as proline, soluble sugar, and soluble protein in alfalfa seedlings with Si pretreatment or without Si pretreatment under alkaline stress. Data showed that alkaline stress remarkably enhanced the accumulation of compatible solutes in alfalfa seedlings. We could see from the **Figure 5**, the soluble sugar, soluble protein and proline content were significantly increased compare with the control under of alkaline stress (*P* < 0.05). However, the treatment with Si priming significantly lowered soluble protein (16.4%) and proline contents (23.1%) (*P* < 0.05) in alfalfa leaves than the treatment without Si pretreatment under alkaline stress, but Si priming had no significant effects on the soluble sugars contents (**Figure 5**).

The changes of osmotic potentials of Si priming-triggered are not only affected by the accumulation profiles of metabolites but are also regulated by the allocation of Na and K in alfalfa under alkaline stress. Therefore, we analyzed the Na and K contents in alfalfa tissues to evaluate the distribution changes of Na and K in alfalfa under Si priming in response to alkaline stress. Data showed that alkaline stress resulted in a decrease of K accumulation in alfalfa roots than the control without alkaline stress (**Table 2**). Both the stems and leaves demonstrated higher K accumulation than the roots in response to alkaline stress or favorable culture (**Table 2**). Data also revealed that Si priming mainly produced higher K accumulation in alfalfa leaves under favorable culture conditions; however, the treatment with Si priming only produced slightly higher K accumulation in all of alfalfa organs under alkaline stress than the treatment without Si priming. In contrast, the pretreatment of Si priming produced lower K accumulation in alfalfa stems, but had higher K accumulation in alfalfa leaves under favorable culture conditions (**Table 2**). Different from the profiles of K accumulation, the treatment of Si priming produced significantly higher accumulation of Na in all tissues under alkaline stress than that of the control under favorable culture conditions. Meanwhile, Si priming led to higher accumulation of Na in the roots under alkaline stress, but decreased accumulation of Na in leaves than the treatment without Si priming.

**Table 2:** Effects of Si priming on the distributions of metal salt ions and nutrient elements in alfalfa seedlings under alkaline stress.

Na_2_CO_3_ (mM)		K (mg g^-1^)	Na (mg g^-1^)	Ca (mg g^-1^)	Mg (mg g^-1^)	Fe (μg g^-1^)	Mn (μg g^-1^)	Cu (μg g^-1^)	Zn (μg g^-1^)	P (mg g^-1^)	S (mg g^-1^)
**Root**											
0	-Si	25.36 ± 2.13a	1.32 ± 0.20d	9.26 ± 0.68b	4.42 ± 0.26ab	1375.88 ± 178.88ab	31.12 ± 3.98a	27.68 ± 0.29b	8.16 ± 0.49ab	7.24 ± 0.40a	3.72 ± 0.20a
	+Si	29.72 ± 4.11a	7.94 ± 0.73c	5.06 ± 0.38c	3.44 ± 0.32b	1706.56 ± 266.21ab	37.20 ± 4.30a	29.46 ± 1.36ab	9.76 ± 0.74a	7.26 ± 0.57a	3.58 ± 0.26a
25	-Si	5.32 ± 0.90b	12.32 ± 1.62b	12.48 ± 0.54a	5.06 ± 0.50a	1981.14 ± 269.41a	38.56 ± 4.83a	33.60 ± 2.26a	7.36 ± 0.93bc	6.48 ± 0.30a	1.88 ± 0.07b
	+Si	5.56 ± 0.46b	16.70 ± 0.59a	13.06 ± 1.25a	3.82 ± 0.37b	1185.08 ± 140.57b	11.22 ± 1.78b	30.50 ± 0.15ab	5.60 ± 0.34c	4.90 ± 0.25b	1.84 ± 0.05b
**Stem**											
0	-Si	45.70 ± 1.42a	0.72 ± 0.10b	11.90 ± 0.67a	2.00 ± 0.17a	100.80 ± 8.69a	3.08 ± 0.41a	42.36 ± 5.60a	10.92 ± 4.94a	3.98 ± 0.15b	2.02 ± 0.10a
	+Si	40.76 ± 2.22a	1.02 ± 0.11b	8.66 ± 0.51b	1.38 ± 0.07b	85.24 ± 7.84a	3.16 ± 0.74a	38.26 ± 8.01ab	5.74 ± 0.93a	4.00 ± 0.23b	1.68 ± 0.11a
25	-Si	39.22 ± 2.59a	16.28 ± 1.44a	8.04 ± 1.16b	1.42 ± 0.18b	99.30 ± 12.28a	1.50 ± 0.45b	27.50 ± 0.29ab	8.02 ± 1.87a	5.18 ± 0.36a	1.94 ± 0.19a
	+Si	39.32 ± 2.47a	15.50 ± 2.33a	8.06 ± 1.17b	1.64 ± 0.22ab	90.02 ± 9.67a	1.20 ± 0.44b	26.02 ± 0.24b	5.18 ± 1.04a	4.90 ± 0.36a	2.00 ± 0.21a
**Leaf**											
0	-Si	44.78 ± 4.28c	0.82 ± 0.17b	17.02 ± 1.19c	3.44 ± 0.11ab	164.48 ± 27.71b	16.62 ± 1.50ab	26.10 ± 0.35a	10.08 ± 0.35a	5.36 ± 0.29b	4.30 ± 0.23b
	+Si	60.60 ± 1.76a	1.04 ± 0.17b	16.90 ± 0.7 4c	3.26 ± 0.17b	140.26 ± 7.91b	13.88 ± 0.67bc	26.50 ± 1.39a	10.30 ± 0.80a	6.08 ± 0.42b	3.98 ± 0.31b
25	-Si	52.28 ± 1.77b	16.60 ± 2.15a	24.32 ± 1.25a	3.78 ± 0.12a	230.12 ± 19.83a	17.32 ± 0.80a	26.38 ± 0.39a	11.32 ± 0.65a	8.22 ± 0.51a	5.08 ± 0.13a
	+Si	54.96 ± 0.24ab	13.50 ± 2.34a	20.46 ± 0.90b	3.68 ± 0.19ab	143.92 ± 18.02b	10.96 ± 0.89c	24.90 ± 0.60a	7.56 ± 0.46b	6.52 ± 0.68b	4.06 ± 0.15b


***-**Si, pretreatment without Si; +Si, pretreatment with Si. Values are means ± SE, *n* = 25. Different letters denote significant difference (*P* < 0.05) based on Duncan’s test.*

Analyses showed that Si pretreatment has a substantial decrease in K/Na ratios in root and stem than the treatments without Si priming under favorable conditions; however, although there was not statistically significant, the K/Na ratio in the shoots of the Si priming treatment was higher (increased by 0.34 in the stems and by 1.50 in the leaves) than those of the treatment without Si priming under alkaline stress (**Table 3**). The partial metal ions and nutrient elements that participated in physiological metabolism were also investigated. Data showed that the accumulation of the metal ions Fe, Mn, and P in the roots and leaves, the Ca, Zn, and S in the leaves, the Mg in the roots were significantly decreased by Si pretreatment under alkaline stress conditions compared to the treatment without Si pretreatment. However, Cu accumulation had no significant changes under Si priming in response to alkaline stress (**Table 2**).

**Table 3:** Effects of Si priming on the changes of K/Na ratio in alfalfa seedlings under alkaline stress.

Na_2_CO_3_ (mM)		Root	Stem	Leaf	Whole plant
0	-Si	20.88 ± 3.54^a^	71.32 ± 14.50^a^	66.06 ± 17.61^a^	44.59 ± 8.51^a^
	+Si	3.84 ± 0.67^b^	41.78 ± 5.82^b^	64.26 ± 8.74^a^	13.28 ± 0.74^b^
25	-Si	0.42 ± 0.05^b^	2.50 ± 0.32^c^	3.36 ± 0.39^b^	2.20 ± 0.19^b^
	+Si	0.32 ± 0.04^b^	2.84 ± 0.52^c^	4.86 ± 1.14^b^	2.33 ± 0.32^b^


*-Si, pretreatment without Si; +Si, pretreatment with Si. Values are means ± SE, *n* = 25. Different letters denote significant difference (*P* < 0.05) based on Duncan’s test.*

## Discussion

Silicon has been widely utilized as an exogenous conditioner and plays a vital role in defending against environmental damage of plants. Different plant species have exhibited diverse requirements for Si addition (Etesami and Jeong, 2018). To obtain the optimum dosage of Si in the present study, we first analyzed the adsorption rates of Si in alfalfa seedlings by culturing in Hoagland’s solutions under the presence of different Si concentrations under alkaline stress of 20 mM Na_2_CO_3_ (Supplementary Figure S1A) or alkaline stress of 25 mM Na_2_CO_3_ (Supplementary Figure S1B). Data revealed that the highest adsorption peak of Si in alfalfa occurred when the 2.25 mM Na_2_SiO_3_ solution with Hoagland components was used to culture plants (Supplementary Figure S1C), thereby determining that 2.25 mM Na_2_SiO_3_ was the optimum concentration for the addition of Si element dosage under alkaline stress. Si treatment at this concentration had no appreciable effects on plant growth and physiological parameters under unstressed conditions (data not shown), indicating that the Si priming effects observed in this study were specific to alkaline stressed plants.

Biomass is one of the reliable indicators of the plants responses to stress, and alkalinity stress obviously inhibits plant’s growth and development (Wei et al., 2017; Zhang et al., 2017). Si plays an important role in increasing biomass by promoting the net Pn under salt stress (Yin et al., 2013). In the present study, alkaline stress significantly inhibited the growth of alfalfa seedlings, and decreased aboveground biomass, and restricted the formation of photosynthetic products, thus resulting in wilting or death of plants. However, Si priming beneficially altered the root morphology of alfalfa seedlings, which enhanced the uptake ability of the roots to nutrients and moisture, and significantly increased root DW and average diameter (**Figure 1**). Enhanced increases in the Pn, Gs, Tr, and WUE in alfalfa leaves (**Table 1**) possibly resulted from an alleviation in the oxidative damage to photosynthetic apparatus under Si priming (Liang, 1998), because accumulation of Na in leaves was reduced, but K accumulation in leaves was increased when alfalfa seedlings were pretreated by Si priming (**Table 2**). In plants, overcoming osmotic stress or physiological water deficit is one of the most important strategies of plant adaptation to salt and/or salinity stressful environments. Earlier studies have shown that Si could improve WUE by depositing keratin-bilayer structures onto the leaf surface or reducing transpiration losses in plants (Guntzer et al., 2012; Shi et al., 2014; Li et al., 2015). However, in the present study, Si priming significantly increased Tr in alfalfa seedlings, showing that it was not the physical deposition that decreased the transpiration water loss and increased WUE as shown in maize under alkaline stress (Abdel Latef and Tran, 2016); therefore, there might be other regulatory approaches that need to be further explored in the transport and storage of water in alfalfa seedlings.

To date, the report on Si priming involved in the protection of the oxidative defense system in alfalfa under alkaline stress is lacking. Studies have confirmed that salt stress usually leads to a loss of balance between the production and removal of reactive oxygen species (ROS), thus resulting in more accumulation of ROS and aggravation of oxidative damage to proteins, nucleic acids, and lipids (Vaidyanathan et al., 2003; Zhu et al., 2015). Excess ROS in plant cells leads to the occurrence of lipid peroxidation and increases more accumulation of MDA (Shi et al., 2014; Zhang et al., 2017). Studies have confirmed that the involvement of Si could lower the concentration of MDA, which is the end-product of lipid peroxidation, in salt-stressed barley (Liang et al., 2003) and sorghum (Liu et al., 2015), thereby maintaining membrane integrity and permeability (Raven, 2001). In the present study, the MI alleviation and the lower accumulation of MDA of Si priming-triggered suggest that Si priming decreased the membrane lipid peroxidation under alkaline stress and might result in a series of physiological responsive mechanisms to mitigate oxidative damage in alfalfa seedlings under alkaline stress, since Si priming is confirmed to be involved in the physiological regulation of salt-stressed barley (Liang et al., 2003). Key antioxidant enzymes e.g., APX, CAT, SOD, and POD, usually play crucial roles in defending against damage of ROS accumulation to the cell membrane system in plants (Limon-Pacheco and Gonsebatt, 2009; Kumar et al., 2017). In the present study, CAT, and POD activities in alfalfa leaves were significantly increased, suggesting that Si priming effectively decreased the permeability of plasma membranes and membrane lipid peroxidation, this might be resulted from lowering the accumulation of ROS, and preventing the structural and functional deterioration of cell membranes in alfalfa under alkaline stress. Meanwhile, Si priming lowered the oxidative damage to the photosynthetic organs in alfalfa, thus increasing the accumulation of chlorophyll in leaves.

In addition, Si priming significantly reduced the accumulation of soluble protein, and proline contents, which might lead to alleviation in photosynthetic feedback repression (Richter et al., 2015; Wei et al., 2015). Generally, the increasing of soluble sugar, soluble protein and proline content is helpful for alleviating the damages under abiotic stress. But in our study, Si priming significantly reduced the accumulation of soluble protein and proline content. It is speculated that there was little relationship between the Si priming mechanism and osmotic regulation of alfalfa seedlings under alkaline stress. The results are in contrast with the observation in maize (Abdel Latef and Tran, 2016). This might partially result from Si priming in promoting the photosynthesis and activating antioxidant defense systems caused by alkaline stress (Vaculík et al., 2015; Zhu et al., 2016). Commonly, the accumulation level of proline in plants is closely associated with the environmental stress degree and plays a functional role in increasing stress tolerance (Boscaiu et al., 2013). The responses of plants to abiotic stresses are based on the synthesis and accumulation of osmolytes in many plants because beneficial accumulation profiles of osmolytes not only play important regulatory roles in maintaining cellular osmotic balance under abiotic environmental stresses (such as drought, salinity, cold or high temperatures), but also function as ‘osmoprotectant’ in the way of low molecular-weight chaperons or ROS scavengers (Vicente et al., 2016). Therefore, our data suggest that Si priming might function through the responsive regulations of osmolytes and activate the antioxidant defense systems in an attempt to cope with cellular damage caused by alkaline stress.

Apart from metabolite allocations, accumulation profiles of metal ions in plants naturally determine the magnitude of salt damage to plants. Wang and Han (2007) reported that exogenous Si significantly reduced the uptake of salt ions in alfalfa roots. Si could alleviate the damage of salinity stress by influencing the translocation of Na and K in plants (Zhu and Gong, 2014; Rizwan et al., 2015). In the present study, Si priming accumulated more Na in the roots and more K in the leaves, and decreased the accumulation of Na in leaves, indicating that Si priming efficiently blocked the transport of toxic ions from the roots to the shoots of the alfalfa, since blocking the transport of toxic ions from the roots to the aerial parts of the plant might greatly contribute to the increasing tolerance of salt stress (Kumar et al., 2017). In addition, Si priming lowered the accumulations of the other metal ions Fe, Mn, and Zn in roots and leaves of alfalfa than the treatment without Si priming under alkaline stress (**Table 2**). This suggests that Si pretreatment primed the alfalfa seedlings for an active role in the ion regulation to alkaline stress. Therefore, further study is required to investigate the regulatory mechanism involved in the osmotic regulation.

## Conclusion

In summary, Si priming altered the accumulation profiles of compatible metabolites and metal salt ions such as Na, Mg, Fe, Mn, and Zn in alfalfa roots, and lowered the ion osmotic damage to roots, thus leading to an increase in Pns and alfalfa biomass in response to alkaline stress. In addition, Si priming increased the activities of antioxidant enzymes in alfalfa seedlings and alleviated the oxidation damage, thus contributing to an enhanced alkaline tolerance in alfalfa. The present study provides evidence for clarifying the physiological modulation of Si priming and the application of exogenous Si in alfalfa planting in soils with high alkalinity.

## Author Contributions

DL performed all the experiments and prepared the first manuscript. ML participated in the physiological and morphological measurements, data analyses and prepared the final manuscript. X-LL participated in the ions measurements. X-GC analyzed the data and corrected the manuscript. Z-WL designed the experiment and supervised the experimental process.

## Conflict of Interest Statement

The authors declare that the research was conducted in the absence of any commercial or financial relationships that could be construed as a potential conflict of interest.
