# Supplementary material for: Silicon Priming Created an Enhanced Tolerance in Alfalfa (Medicago sativa L.) Seedlings in Response to High Alkaline Stress
Source: Front Plant Sci. 2018 May 29;9:716. doi: 10.3389/fpls.2018.00716 (PMC5986902; doi:10.3389/fpls.2018.00716)
Supplement: Supplementary file 1 [file Image_1.pdf]

Supplementary Material

Silicon Priming Created an Enhanced Tolerance in Alfalfa (*Medicago sativa* L.) Seedlings in Response to High Alkaline Stress

Liu et al.
FIGURE S1

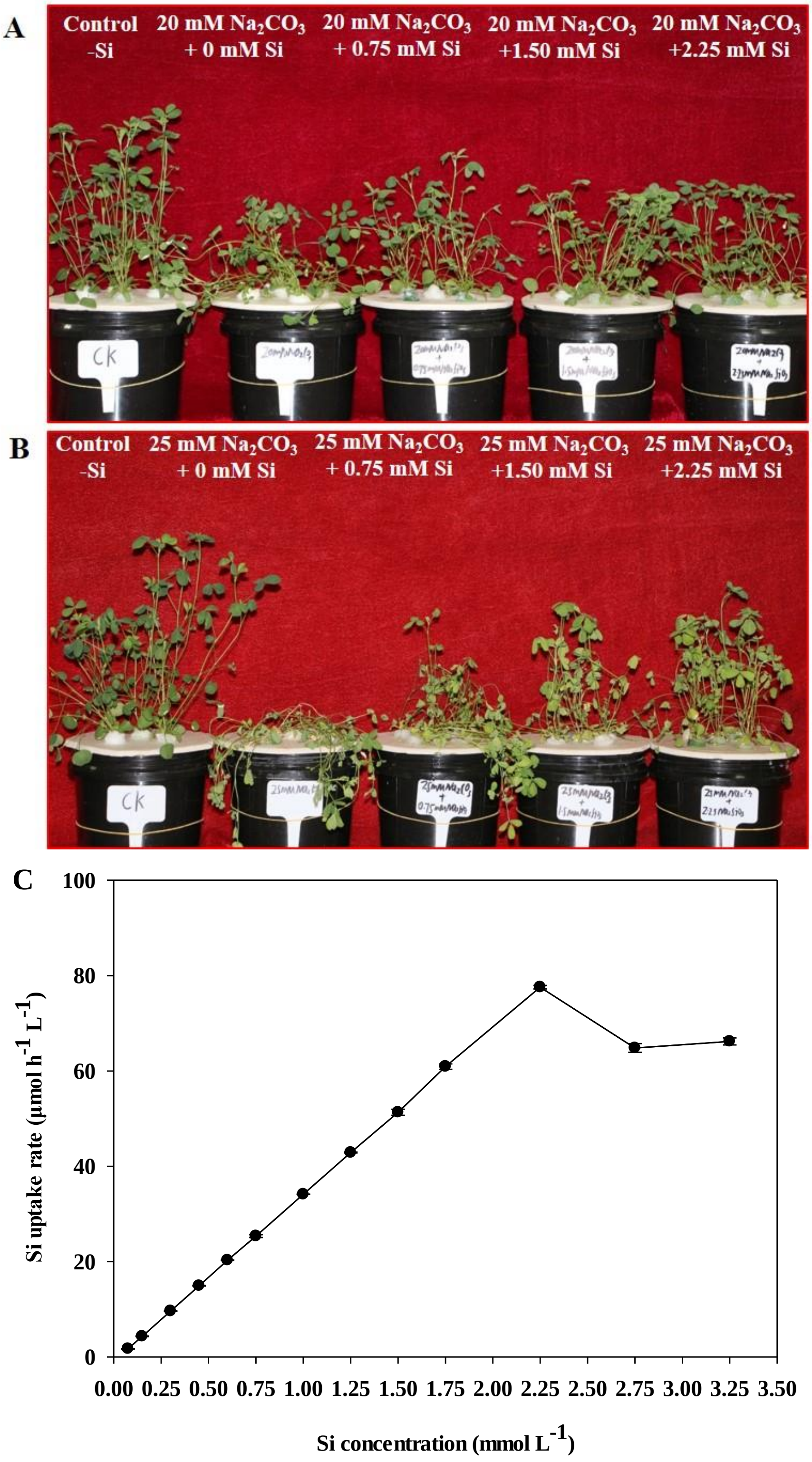

FIGURE S1 | Adsorption rates of silicon in alfalfa in the presence of Si. (A) The effects of Si priming under alkaline stress of 20 mM Na_2CO_3, (B) The effects of Si priming under alkaline stress of 25 mM Na_2CO_3, (C) Adsorption rates of Si in alfalfa in the presences of different Si level.
